# The Protein Tyrosine Phosphatase H1 PTPH1 Supports Proliferation of Keratinocytes and is a Target of the Human Papillomavirus Type 8 E6 Oncogene

**DOI:** 10.3390/cells8030244

**Published:** 2019-03-14

**Authors:** Stefanie Taute, Philipp Böhnke, Jasmin Sprissler, Stephanie Buchholz, Martin Hufbauer, Baki Akgül, Gertrud Steger

**Affiliations:** 1 Institute of Virology, University of Cologne, Faculty of Medicine and University Hospital of Cologne, Fürst-Pückler-Strasse 56, 50935 Cologne, Germany; Steffi.taute@me.com (S.T.); pboehnke@mn-net.com (P.B.); Jasmin.sprissler@uni-ulm.de (J.S.); stephanie.buchholz@bfarm.de (S.B.); martin.hufbauer@uk-koeln.de (M.H.); baki.akguel@uk-koeln.de (B.A.); 2 ViiV Healthcare, Prinzregentenplatz 9, 81675 Munich, Germany; 3 Macherey-Nagel GmbH&Co. KG, Neumann Neander Str. 6-8, 52313 Düren, Germany; 4 Institute for Clinical Transfusion Medicine and Immunogenetics Ulm, Helmholtzstr. 10, 89081 Ulm, Germany; 5 Federal Institute for Drugs and Medical Devices BfArM, Kurt-Georg Kiesinger Allee 3, 53175 Bonn, Germany

**Keywords:** Human papillomaviruses (HPV), HPV8, E6 oncoprotein, protein tyrosine phosphatase H1 (PTPH1), EGFR, active Ras, keratinocyte proliferation

## Abstract

Human papillomaviruses (HPV) replicate their DNA in the suprabasal layer of the infected mucosa or skin. In order to create a suitable environment for vegetative viral DNA replication HPV delay differentiation and sustain keratinocyte proliferation that can lead to hyperplasia. The mechanism underlying cell growth stimulation is not well characterized. Here, we show that the E6 oncoprotein of the βHPV type 8 (HPV8), which infects the cutaneous skin and is associated with skin cancer in Epidermodysplasia verruciformis patients and immunosuppressed organ transplant recipients, binds to the protein tyrosine phosphatase H1 (PTPH1), which resulted in increased protein expression and phosphatase activity of PTPH1. Suppression of PTPH1 in immortalized keratinocytes reduced cell proliferation as well as the level of epidermal growth factor receptor (EGFR). Furthermore, we report that HPV8E6 expressing keratinocytes have increased level of active, GTP-bound Ras. This effect was independent of PTPH1. Therefore, HPV8E6-mediated targeting of PTPH1 might result in higher level of EGFR and enhanced keratinocyte proliferation. The HPV8E6-mediated stimulation of Ras may be an additional step to induce cell growth. Our results provide novel insights into the mechanism how βHPVE6 proteins support proliferation of infected keratinocytes, thus creating an environment with increased risk of development of skin cancer particularly upon UV-induced DNA mutations.

## 1. Introduction 

Infection with human papillomaviruses belonging to the genus beta-papillomavirus (βHPV), which exhibit a tropism for the cutaneous epithelium, occurs early in life. The viruses replicate in the hair follicles with no apparent lesions in the skin. However, persistent infections with certain types of βHPVs are associated with the development of non-melanoma skin cancer at sun-exposed sites. In particularly, patients with the rare disease epidermodysplasia verruciformis suffer from βHPV-mediated skin carcinogenesis (reviewed in Reference [1]). There is also a direct link between βHPV infection and skin cancer development in immunosuppressed organ transplant recipients (OTR), who have a 100-fold increased risk to develop cutaneous squamous cell carcinomas (cSCC) [2] and a 250-fold increased risk for solar precancerous actinic keratosis (AK) [3,4]. Iatrogenic immunosuppression in OTR allows a more active replication of the commensal βHPV spectrum in the entire skin with the result of higher viral load [2,5]. A contribution of βHPVs in SCC in the normal immunocompetent population remains elusive. UV light is an important co-factor for skin cancer development. UV light-induced DNA mutations in oncogenes and tumor suppressor genes promote the progression of AK to cSCC [6]. 

The oncogenic activity of βHPV regulatory proteins has been demonstrated in mouse models. The expression of βHPV early E6, E7 and E2 genes in the murine skin leads to tumor formation [7,8,9,10,11,12]. However, the primary function of the HPV oncogenes is to create an environment, which allows vegetative viral DNA replication. As proposed for genital HPV types, the initial HPV infection and viral genome maintenance occurs in basal stem cells rather than in transiently amplifying cells. The productive phase of the viral life cycle, including DNA replication, late gene expression and virion production, is initiated upon epithelial differentiation. The presence of the E6 and E7 proteins in the upper epithelial layers allows the differentiating keratinocytes to reenter S-phase, while expressing markers of keratinocyte differentiation [13]. This leads to a delay in keratinocyte differentiation and sustained proliferation resulting in epidermal hyperplasia. 

In the case of the βHPV type 8 (HPV8) it has been shown that E6 is a major oncogene in the murine skin and HPV8E6 cooperates with UV-light in skin tumor induction [12,14]. E6 oncoproteins of βHPV are able to suppress UV-induced apoptosis and DNA repair by degrading the pro-apoptotic Bcl2 family member Bak [15,16] and by delaying UV-induced DNA damage signaling of the ATM/ATR kinases in keratinocytes [17,18]. The prevention of apoptosis by βHPVE6 proteins privileges keratinocytes with UV-induced mutations to survive [14]. Furthermore, βHPV E6 proteins target the Notch co-activator mastermind-like protein 1 (MAML1), and thereby inhibit Notch dependent transcription [19,20]. Notch signaling is an important driver of keratinocyte differentiation. The mechanism underlying the ability to support proliferation and extend the life span of human foreskin keratinocytes as shown for E6 of HPV8 and other βHPV types [17,21] is not yet entirely solved. 

We have previously shown that HPV8E6 stimulates the receptor-tyrosine kinase (RTK) activity of the epidermal growth factor receptor (EGFR) in response to UV-irradiation. This activity by HPV8E6 was crucial for the UV-induced development of skin papillomas in K14-HPV8E6 transgenic mice indicating that E6 enhances the effect of UV-light at the level of EGFR [22]. Our earlier work also showed that HPVE6 proteins can bind to the protein-tyrosine-phosphatase H1 (PTPH1) [23]. PTPH1 (gene name: PTPN3) and the closely related PTPN4 (PTPMEG) are non-transmembrane protein tyrosine phosphatases (PTPs) that contain an N-terminal FERM (Band4.1, Ezrin, Radixin, Moesin homology) domain followed by a single PDZ (PSD95, Dlg, ZO-1) domain and the C-terminal catalytic PTP domain [24]. PTPH1 was described to control cell growth, proliferation and development of cancer of colon, breast and gliomas [25,26,27,28,29,30,31,32]. 

PTPH1 activity has been implicated in EGFR and Ras signaling [33], which are both crucial for cSCC pathology. The EGFR plays an essential role in epidermal homeostasis maintenance as activating EGFR-ligands (EGF, TGF-α and IGF) promote keratinocyte proliferation whereas inhibitory ligands (Lrig1 and Mig6) maintain stem cells quiescence. EGFR is also activated by UV-light, which increases keratinocyte proliferation, suppresses apoptosis, and accelerates epidermal hyperplasia [34,35]. Constitutive activation of EGFR is common in cancer due to mutations or overexpression. Normally, ligand-binding to EGFR results in receptor dimerization and stimulation of the RTK activity which autophosphorylates a number of intracellular tyrosine residues and cytoplasmic substrates. These phospho-tyrosine residues are binding sites for a variety of adapter molecules mediating the stimulation of canonical downstream pathways such as the Ras/Raf/MEK/ERK and PI3K/Akt pathways, while the binding of the E3 ubiquitin ligase Cbl initiates endocytotic internalization, ubiquitinylation and proteasomal degradation of the EGFR to terminate the signaling. In breast cancer cells, PTPH1 was shown to catalyze EGFR desphosphorylation of the phospho-tyrosine in pos. 1173 (pY1173-EGFR) thereby increasing the stability of the EGFR [36]. PTPH1 was also found to cooperate with activated Ras in oncogenic activities in colon cancer cells (reviewed in Reference [33]). Activating mutations in codons 12, 13 or 61 of Ras genes have been detected in at least 22% of human SCCs, and in experimentally induced cSCC in mouse models. However, the number of human tumors with a constitutively active Ras pathway might be significantly higher (reviewed in [37]). Ras signaling is essential for proper development of the epidermis and hair follicles. In vivo experiments indicate that Ras promotes epidermal proliferation and inhibits differentiation [38]. Constitutive activation of Ras in keratinocytes does not lead to transformation. However, activation of Ras by mutations or upstream signaling pathways is regarded as an early event in skin tumor formation. Two genetic events, i.e., K- Ras induction followed by TP53 inactivation, as consequence of UV irradiation [39] are necessary for progression from papilloma to SCC [37]. 

We and others have reported previously, that E6 from the high-risk αHPV types HPV16 and HPV18 target PTPH1 via PDZ-mediated interaction which resulted in the accelerated proteasomal degradation of PTPH1 [23,40]. However, no evidence for a physiological role of this interaction in the oncogenic activity of HPV16E6 was found [41]. In addition, our results showed that the binding of the HPV8 E6, which does not encode a PDZ-binding motif, did not induce the degradation of recombinant PTPH1 [23]. Here we report that HPV8E6 increases the amounts of PTPH1. We demonstrate that PTPH1 supports proliferation of keratinocytes and regulates the EGFR. In addition, HPV8E6 expressing keratinocytes have increased level of active GTP-bound Ras. Our results suggest that targeting of PTPH1 and activation of Ras by HPV8E6 mediates sustained keratinocyte proliferation and might thus create an environment with increased risk of the development of skin cancer upon UV-induced DNA mutation.

## 2. Materials and Methods 

### 2.1. Cell Culture 

Normal human epidermal keratinocytes (NHEK) were obtained from PromoCell and were cultivated in KGM2 (PromoCell) supplemented with growth factors and penicillin and streptomycin (P/S). HaCaT (provided by Professor N. Fusenig, German Cancer Research Center, Heidelberg, Germany; CLS Cell Lines Service, 300493) [42] and C33A cells (obtained from ATCC) were grown in Dulbecco’s Modified Eagle Medium (DMEM). The immortalized keratinocyte cell lines RTS3b (obtained from I. Leigh [43]) and N/TERT (obtained from the ATCC), were cultivated in E-Medium [44]. DMEM and E-Medium were supplemented with 10% fetal bovine serum (FBS) and P/S. Transduction with pLXSN or pLXSN-HPV8E6 and selection by G418 was performed according to a previous protocol [45]. RTS3b-8E6 and empty vector cells were transfected by FuGENE6 transfection reagent (Promega) followed by selection with G418 and puromycin. C33A cells were transfected with the calcium phosphate method, and siRNAs were transfected with Hiperfect (Qiagen). UV irradiation was performed at a dosage of 30 or 40 mJ/cm^2^ UVB using a UVP CL-1000 ultra-violet cross-linker with F8T5 bulbs giving a spectral peak a 320 nm for 10 s. 

### 2.2. Plasmids, siRNA 

The expression vectors pLXSN and pLXSN-HPV8E6 have been described previously [46]. Myc-PTPH1, pcDNA-FLAG empty vector (EV), pXJ41-FLAG-8E6 or pXJ41-FLAG-16E6 are described in [23], Myc-NAP1 in [47] and plasmids for Myc-PTPH1 deletion mutants and GST-PTPH1 were obtained by cloning appropriate PCR products. Point mutations were introduced into the pXJ41-FLAG-8E6 plasmid by site directed in vitro mutagenesis. FLAG-p38γ was kindly provided by D. Livnah [48]. A PCR product encoding the minimal Ras binding domain of Raf was cloned into pGEX2T. The siRNA against HPV8E6 was described in [49]. SiRNAs against PTPH1 (M-009372-01) and p38γ (MAPK12) (M-003590-03) as well as non-coding control siRNA (D-001 206 1420) were from Dharmacon. pGIPZ non-coding control (cat-no. RHS4346) and pGIPZ PTPN3 shRNA plasmids (RHS4531-EG5774) were purchased from Thermo Scientific. 

### 2.3. RNA-Isolation, RT-PCRs

RNAs were isolated by the Nucleo Spin RNA Plus kit from Macherey-Nagel. RT-PCR was performed with the GoScript Reverse Transcriptase (A5004; Promega) and random primers, and qPCR with the GoTaq-qPCR SYBRGreen master mix (A6002, Promega) and gene specific primers, with the sequences provided in Supplementary Table S1, in a Light Cycler 480 (Roche Diagnostics). Transcripts for PTPH1, p38γ/MAPK12, EGFR and HPRT were amplified with the GoTaq probe master mix (A6101, Promega) and detected by UPL-probes (Roche Diagnostics). 

### 2.4. Antibodies

Antibodies against PTPH1 used in Western blots (WBs) were from Santa Cruz, (Sc-9789), Abgent (AP8426a) or was a monoclonal antibody (2-117) kindly provided by N. Tonks [50]. The antibodies against the EGFR (D38B1), the pY1068-EGFR (D7A5) and pY1173-EGFR (53A5), pp38MAPK (Thr182/Tyr182, #9211), Myc (71D10), as well as the rabbit IgG isotype control (DA1E) were from Cell Signaling. The anti-Pan-Ras RAS10 (MABS195) antibody was from MerckMillipore. The antibody against Eps15 (no. 610806) was from BD Bioscience. Magnetic M2-FLAG-affinity gel and the FLAG-M2 antibody were from Sigma. 

### 2.5. Western Blots, Co-Immunoprecipitation (Co-IP) and GST-Pull Down Assays

Cells were washed twice in ice cold PBS and scraped in low salt lysis buffer (LSDB) (20% glycerol, 50 mM Tris pH 7.9, 100 mM KCl, 0.1%Nonidet-P40, 1 mM DTT) supplemented with protease inhibitors PMSF, Aprotinin, Leupeptin and Pepstatin and the phosphatase inhibitors NaF and Na-Orthovanadate on ice followed by sonification. IPs, WB as well as GST-pull down assays were done according to standard procedures. Blots were either exposed with X-Ray films and scanned to digitalize or with the ChemiDocXRS System (Bio-Rad). These images were quantitated by the ImageJ software. Some blots were developed and quantitated by the Odyssey system (Li-Cor Biosciences). 

### 2.6. BrdU- and Proliferation Assays

Forty h after transfection, cells were incubated with 10 µM BrdU for 90 min. The BrdU assay was performed with the BrdU-Flow Kit from BD Bioscience (Cat.no. 559619) and quantitated by FACS analysis. The final analysis was done with the FlowJo software. RTS3b-8E6 shGIPZ control or shPTPN3#5 cells were seeded in six-well plates in triplicates. Cells were trypsinized after different time points and counted by a TC10 automated cell counter (BioRad). 

### 2.7. Immunostainings 

Tumors that appeared in K14-HPV8E2 transgenic mice [8] or 3 weeks after UV-irradiation in K14-HPV8E6 transgenic mice [12] were excised, fixed in 4% paraformaldehyd and processed for paraffin embedding. IHC staining was performed with the anti-PTPH1 antibody (AP8426a) from Abgent and the Vectastain Universal Elite ABC Kit (Vector Laboratories) and counterstained with hematoxylin/eosin (H&E) and Giemsa following standard protocols. Sections were analyzed with the Leica DM4000B light microscope equipped with a KY-F75U digital camera (JVC) and Diskus 4.50 Software. 

### 2.8. PTP-Assay

The phosphatase assay was performed with the Malachite Green PTP-assay kit 1 from Upstate (17-125, Merck) according to the instructions of the kit. Briefly, 48 h after transfection of C33A cells, total extracts were incubated with Myc-beads overnight at 4 °C. After several washing steps the pellet was resuspended in 50 µL 500 mM pY-peptide (RRLIEDAE(pY)AAR) (provided by the kit) or Growth Hormone Receptor peptide (NFLMDNA(pY)FCEADAKK), followed by 1 h incubation at 30 °C. 100 µL of a Malachite Green solution was added and the OD_620nm_ was measured 15 min later. Lysate of non-transfected cells was included as negative control. A dilution of phosphate was used as standard. The amount of free phosphate was calculated according instructions of the kit. 

### 2.9. Statistics

Statistical analysis was done with the use of Mircrosoft Excel. In each case the fold differences were calculated from at least 3 or more experiments, as indicated in the figures legends. The standard deviations of the means are given in the graphs. To determine significant differences the students *t*-test was applied. The *p*-values are indicated as * (*p* < 0.05) or ** (*p* < 0.01). 

### 2.10. Ethic Statement 

The transgenic mice used in this study have been described previously [8,12]. UV irradiation protocols were approved by the governmental animal care office North-Rhine-Westphalia (Leibnizstraße 10, 45659 Recklinghausen, protocol no. 8.87–50.10.35.08.163) and were in accordance with the German Animal Welfare Act as well as the German Regulation for the protection of animals used for experimental purposes. For UV treatment, age (5 weeks) and sex matched mice were shaved and irradiated once with 10 J/cm^2^UVA and 1 J/cm^2^UVB on a 4 cm^2^ sized dorsal caudal area. All offspring were macroscopically examined regularly for the presence of skin lesions. The animals were sacrificed and samples of the skin were collected, fixed and subsequently embedded in paraffin.

## 3. Results 

### 3.1. Increased PTPH1 Level in HPV8E6 Expressing Keratinocytes 

Data published by the human protein atlas reveal that PTPH1 has a mixed expression pattern with a moderate cytoplasmic positivity in most normal tissues, including keratinocytes, melanocytes and Langerhans cells in normal human skin and low expression in fibroblasts. In addition, moderate PTPH1 expression was detected in 5 out of 6 tested cSCC and in 1 out of 6 basal cell carcinomas (BCC). RNA expression data were not consistent with data obtained by antibody staining which may be indicative for regulation at the protein level (https://www.proteinatlas.org). These observations demonstrate that PTPH1 is expressed in skin and cSCC. 

**Figure 1:**
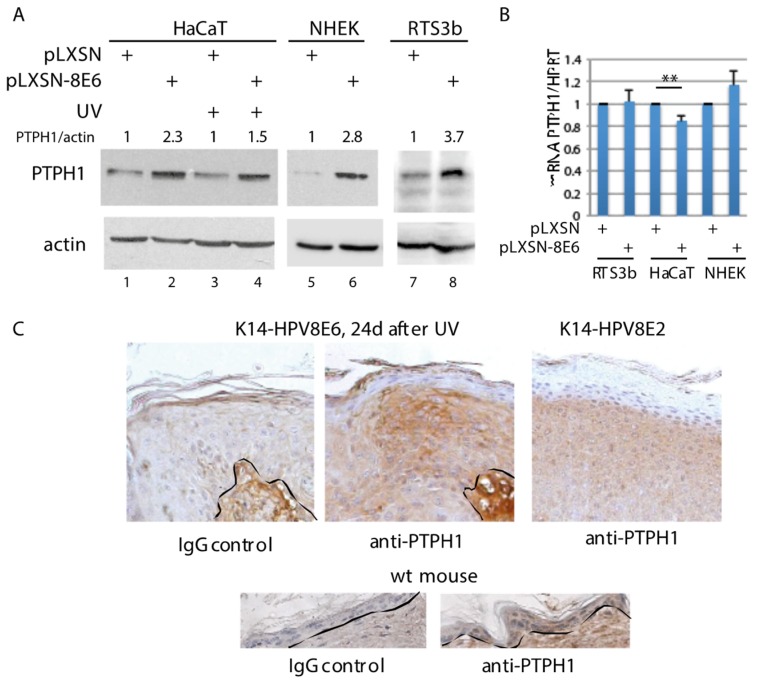
HPV8E6 expressing keratinocyte have increased level of PTPH1. (**A**) Extracts from RTS3b, HaCaT and NHEK containing pLXSN-8E6 or the empty vector were used for WB with an antibody against PTPH1. The cells analyzed in lanes 3, 4 were UV irradiated. The ratios of PTPH1 normalized to actin from the blots shown are given. (**B**) RNA was used for qRT-PCR with PTPH1 and HPRT specific primers. The fold differences were calculated by the comparative ΔΔ threshold method described by Pfaffl [51] (n = 3) (** *p* < 0.01). The standard deviations of the means from 3 independent experiments are included. (**C**) Skin sections from K14-HPV8E6, K14-HPV8E2 transgenic mice and wt mice, were stained with an antibody against PTPH1 or normal rabbit IgG.

Previously we have shown that HPV8E6 targets recombinant PTPH1 without inducing its degradation [23]. Now we extended these studies and analyzed an effect of HPV8E6 on the level of endogenous PTPH1 present in various HPV8E6 expressing immortalized human keratinocyte cell lines and in NHEK. All these keratinocytes have been transduced with recombinant retroviruses expressing HPV8E6 or empty vector pLXSN [22]. Immunoblotting revealed that HaCaT, RTS3b as well as NHEK had higher amounts of endogenous PTPH1 when HPV8E6 was expressed (Figure 1A). The HPV8E6-mediated increase of PTPH1 was not affected by UV-irradiation (Figure 1A, lanes 3, 4). RT-PCR showed that there was no difference in the mRNA level of PTPH1 between empty vector and E6 expressing HaCaT, RTS3b and NHEK cells (Figure 1B). Since we were not able to determine the protein expression of HPV8E6 in these cell extracts due to low expression on one side and to low affinity of antibodies on the other side, the expression of HPV8E6 was confirmed by RT-PCR in all cases (Supplementary Table S2). 

The oncogenic activity of HPV8 and the cooperation with UV-light could be demonstrated in transgenic mouse models which had been established previously in our lab [7,12,49]. The expression of HPV8E6 under control of the keratin 14 promoter (K14-HPV8E6), targeting the expression to basal layer of the squamous epithelium, induced skin tumors within 3 weeks after UV-irradiation [12]. K14-HPV8E2 mice spontaneously developed ulcerous lesions of the skin which mostly appeared as infundibular hyperplasia and acanthosis combined with low-grade dysplasia [8]. To confirm an effect of HPV8E6 on PTPH1 in vivo we used sections of skin tumors from transgenic K14-HPV8E6 or, as control, K14-HPV8E2 mice. A defined PTPH1 specific staining was observed in the proliferating part of the skin tumor, which appeared 24 d after UV-irradiation in K14-HPV8E6 mice. K14-HPV8E2 tumors also expressed PTPH1 but the intensity of the staining appeared weaker, and more homogenous within the single cells. PTPH1 signals were present in the epidermal layers of skin from wild-type (wt) mice as well (Figure 1C). Therefore, also in murine skin tumor tissue, the presence of HPV8E6 correlates with higher amounts of PTPH1. These data support the notion that HPV8E6 increases the endogenous level of PTPH1 via a post-transcriptional mechanism. 

### 3.2. HPV8E6 Directly Targets PTPH1 and Does Not Interfere with the Phosphatase Activity of PTPH1

**Figure 2:**
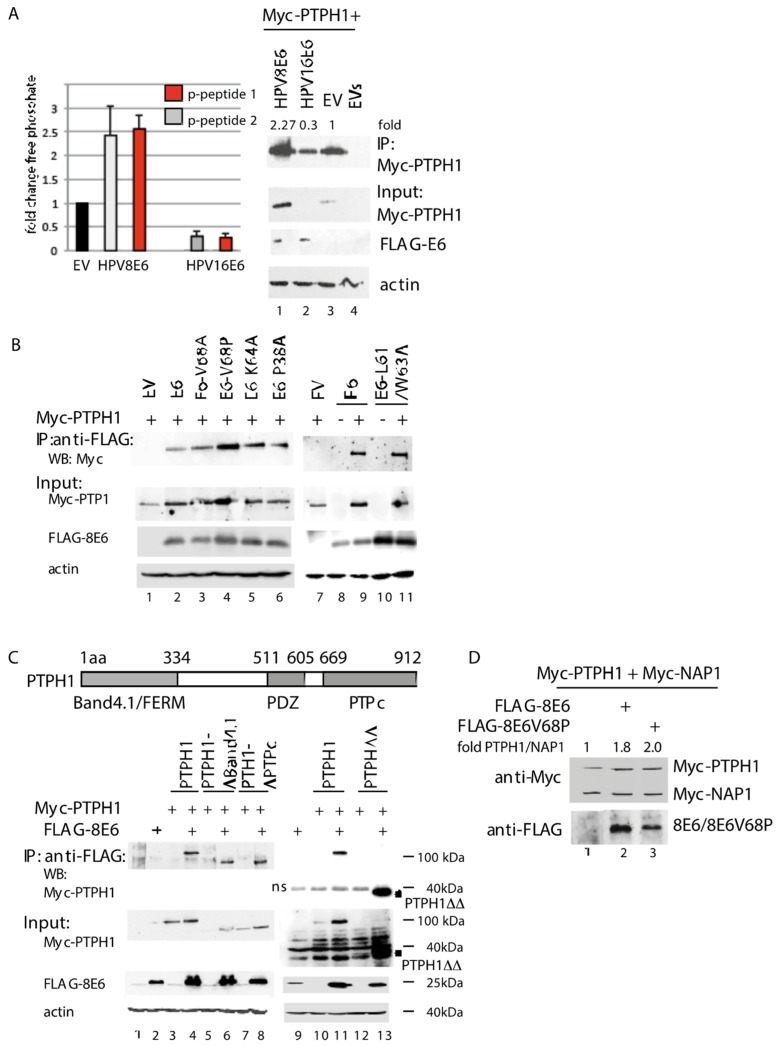
Increased level of PTPH1 in the presence of HPV8E6. (**A**) C33A cells were transfected with expression vectors for Myc-PTPH1 and pcDNA-FLAG empty vector (EV), pcDNA-FLAG-8E6 or pcDNA-FLAG-16E6. The precipitated Myc-PTPH1 was used in a Malachite Green PTP assay according to the manufacturer’s instruction. The values obtained for Myc-PTPH1 without co-expression of an E6 protein was set as 1 and those upon co-expression of E6 of HPV16 and HPV8 have been calculated. The graph represents the results from 3 independent assays. The WB shows the amount of precipitated Myc-PTPH1 which was used for one representative PTP assay, and that in the input, including the FLAG-E6 proteins. The signals were quantified by ImageJ. (**B**,**C**) C33A cells were co-transfected with expression vector for wt Myc-PTPH1 or deletion mutants and pXJ41-FLAG-HPV8E6 wt or point mutants as indicated in the figure. Extracts were incubated with FLAG beads and co-precipitated Myc-PTPH1 was detected by WB. The input was analyzed for Myc-PTPH1 and FLAG-HPV8E6. Actin served as loading control. * indicates the presence of Myc-PTPH1ΔΔ (aa334-669) in the input. In (**C**) an overview of the structure of PTPH1 is given with the localization of the Band4.1/FERM, the PDZ and the C-terminal catalytic PTPc domain. The numbers above refer to the positions of the amino acids (aa). (**D**) Extracts from C33A cells transiently co-transfected with CMV-driven expression vectors for Myc-PTPH1 or Myc-NAP-1 and FLAGHPV8E6 or E6V68P were used for WB to detect the proteins as indicated.

The targeting of PTPH1 by the E6 oncoproteins of the mucosal high-risk HPV types 16 and 18 resulted in the accelerated degradation of PTPH1, thus reducing the intracellular phosphatase activity [19,39]. The interaction of PTPH1 with HPV8E6 neither required the PDZ domain of PTPH1 nor did it induce the degradation of PTPH1 [23]. Nevertheless, HPV8E6 may interfere with the phosphatase activity of PTPH1. In order to test this, we transfected the HPV negative cervical cancer cell line C33A with expression vectors for Myc-tagged PTPH1 and FLAG-tagged HPV8E6 or empty FLAG vector. As control we included an expression vector for FLAG-HPV16E6. Myc-PTPH1 was precipitated by Myc-beads and incubated with two different phospho-tyrosine peptides. One derived from a PTPB1 substrate and the other from the growth hormone receptor, a class I cytokine receptor, which was shown to be dephosphorylated by PTPH1 [50]. The release of free phosphate was quantitated by a Malachite Green assay. With both phospho-tyrosine peptides we observed 2.5-fold higher Myc-PTPH1-phosphatase activity upon co-expression of HPV8E6 compared to co-transfection of the empty FLAG control vector (Figure 2A). Co-expression of HPV16E6 reduced the phosphatase activity by 70%. A WB with the input extracts as well as the Myc-PTPH1-precipitate, which has been used for one of the replicative phosphatase assays, showed that HPV8E6 increased the level of Myc-PTPH1 by 2.3-fold compared to the empty vector while HPV16E6 reduced the PTPH1 level to 0.3-fold. These values perfectly correlated with the amount of the respective phosphatase activities. From these data we conclude that HPV8E6 does not affect the intrinsic enzymatic activity per se but increases the phosphatase activity merely by increasing the level of PTPH1. The Western blot shown in Figure 2B, confirms this notion since the amounts of Myc-PTPH1 were constantly higher when HPV8E6 was co-expressed (Figure 2B, input Myc-PTPH1, lane 1 vs. 2, lane 7 vs. 8). To characterize a role of PTPH1 for known HPV8E6 activities we analyzed the contribution of previously characterized amino acids of HPV8E6 to the interaction with PTPH1. We focused on amino acids within the N-terminal moiety since our previous work showed that this segment of HPV8E6 mediated the binding to PTPH1 while the C-terminal moiety failed to bind [23]. The K64A mutant of HPV8E6 was largely reduced in its ability to precipitate MAML1 as shown previously [20]. Figure 2B reveals that HPV8E6K64A was not impaired in its interaction with PTPH1 indicating that targeting PTPH1 and MAML1 are distinct activities. The P40A, V70A and L63A/W65A mutants in HPV5E6, which correspond to P38A, L61A/W63A and V68A in HPV8E6, respectively, lost completely their activity to induce the degradation of Bak, but still partially inhibited apoptosis [52], indicating that other activities than targeting Bak may contribute to HV8E6-mediated inhibition of UV-induced apoptosis. The HPV8E6 point mutants P38A, L61/W63A and V68A, respectively, were not affected in its ability to precipitate PTPH1 (Figure 2B). Therefore, binding of HPV8E6 to PTPH1 is distinct form that to Bak. 

To further characterize the interaction, we mapped the region in PTPH1 which is targeted by HPV8E6. Figure 2C shows that Myc-tagged PTPH1 deletion mutants lacking either the N-terminal FERM/Band4.1 or the C-terminal PTPc domain were precipitated by FLAG-HPV8E6 in a similar manner than full length Myc-PTPH1. In correlation, HPV8E6 precipitated a mutant PTPH1ΔBand4.1ΔPTPc encoding the central amino acids 334–669 (PTPH1ΔΔ). Intriguingly, this central fragment of PTPH1 was hardly visible in the input blot in the absence of HPV8E6 while it was present in high amounts upon co-expression of HPV8E6. This may reflect the E6-induced stabilization mediated by a direct binding (Figure 2C). We also thought to test for binding of HPV8E6 to the PTPc domain. However, we were neither able to detect the expression of recombinant PTPc by Western blotting, nor to co-precipitate PTPc by HPV8E6 (own unpublished observations). From these data we conclude that HPV8E6 targets the central domain which correlates with higher protein level, although we cannot certainly exclude that the catalytic domain is bound as well. 

The WB shown in Figure 2D reveals that upon the co-expression of HPV8E6 or HPV8E6V68P, the level of Myc-PTPH1 were elevated in contrast to that of the HPV8E6 unrelated protein Myc-NAP1. Myc-NAP-1 and Myc-PTPH1 are both produced from the CMV promoter. These data are in line with the notion that HPV8E6 stabilizes PTPH1. 

### 3.3. PTPH1 Supports Cell Growth and Proliferation in HPV8E6 Expressing Keratinocytes

**Figure 3:**
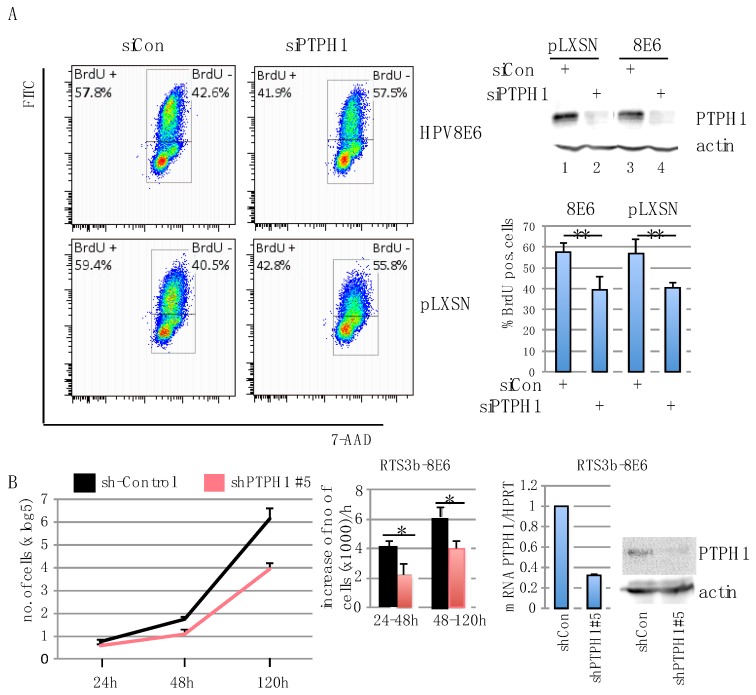
PTPH1 supports proliferation of keratinocytes. (**A**) HaCaT-pLXSN and HaCaT-HPV8E6 cells were transfected with siRNA against PTPH1 or control and used for a BrdU assay. The efficiency of the knock down of PTPH1 was confirmed by Western blot (Lanes 1–4). The graph represents the mean values of BrdU positive cells in % of three independent experiments (** *p* < 0.01) (**B**) RTS3b-E6 cells were transfected with pGIPZ-sh-control or pGIPZ-shPTPH1#5, neomycin and puromycin resistant cells were pooled. RNA and protein extracts were prepared to demonstrate the efficiency of the knock down by RT-PCR and Western blot. 5 × 10^4^ cells were seeded in triplicate in 6-well plates and the number of cells was counted at different time points (* *p* < 0.05).

What might be the consequences of the higher level of PTPH1 in HPV8E6 expressing cells? βHPVE6 proteins were demonstrated to counteract UV-induced apoptosis and impair differentiation and sustain keratinocyte proliferation [1,20]. In agreement with the latter property we observed that NHEKs transduced with pLXSN-8E6 could be cultivated on average for 56 days (+/−7 days) while empty vector cells failed to reattach to the culture plate after trypsinizing in average after 33 d (+/−6 days) (*p* < 0.5) (Supplementary Figure S1). Since PTPH1 was shown to regulate proliferation of various cancer cell lines, we investigated the effect of PTPH1 on proliferation of keratinocytes. We suppressed PTPH1 in HPV8E6 expressing and pLXSN-control HaCaT cells by transiently transfecting a pool of four siRNAs targeting PTPH1 or non-targeting siRNAs as control. Cellular DNA replication was assessed by a BrdU assay followed by FACS analysis. As shown in Figure 3A, HaCaT cells, irrespective of whether they contain HPV8E6 or the empty vector incorporated about 15% less BrdU into their DNA after transfection of the PTPH1 specific siRNAs compared to control siRNA. The efficiency of the knock down was confirmed by Western blotting in both cell lines (Figure 3A). We set up to confirm these results with RTS3b cells. However, here the BrdU-antibody to detect the incorporation of BrdU revealed nonspecific binding, making these experiments hard to interpret. To investigate an effect of PTPH1 on the proliferation of RTS3b cells, we used pGIPZ-lentiviral shRNA constructs to reduce the expression of PTPH1. RTS3b-pLXSN and HPV8E6 cells were transfected with 5 different PTPH1-sh-RNA encoding plasmids and a sh-control construct individually and successfully transfected cells were selected by puromycin. Only the shPTPH1#5 construct gave rise to a robust inhibition of PTPH1 protein expression (data not shown). RTS3b-8E6 shPTPH1#5 cells had about 70% less PTPH1 mRNA compared to shControl cell, and reduced protein level, as demonstrated by RT-PCR and WB (Figure 3B). Since the RTS3b-pLXSNshPTPH1#5 cells hardly grew, only RTS3b-8E6 sh-control and -shPTPH1#5 cells were seeded and changes in the cell number were determined after 24 h, 48 h and 120 h. Figure 3B shows that RTS3b-8E6 shPTPH1#5 had reduced proliferation rate compared to RTS3b-8E6 shControl cells. 24 h to 48 h after seeding the number of control cells increased by 4000 cells/h while that of the PTPH1-sh#5 cells increased only by 2000 cells/h during this time (Figure 3B). These results suggest that PTPH1 supports active DNA replication and proliferation in HaCaT and RTS3b keratinocytes. 

HPV38E6 was shown to increase the proliferation of late passage keratinocytes as well [53]. But, we did not detect increased level of PTPH1 in RTS3b cells expressing HPV38E6 (Supplementary Figure S3). However, RT-PCR revealed that the HPV38E6 mRNA level was about 100-fold lower compared to that of HPV8E6 in RTS3b cells (Supplementary Table S2). Therefore, it cannot be excluded that the amount of HPV38E6 was too low to have an effect on PTPH1.

### 3.4. HPV8E6 Leads to Higher Level of GTP-Ras 

We next thought to address the mechanism how PTPH1 supports proliferation in immortalized keratinocytes. PTPH1 was described as a specific phosphatase of p38γ (mitogen activated protein kinase MAPK12) and vice versa, p38γ phosphorylated PTPH1 at S459 [54,55]. p38γ is a member of the MAPK with a unique C-terminal PDZ-binding motif. Like the other p38 MAPK family members p38α, and β, p38γ is phosphorylated in response to stress, such as UV-irradiation. However, whereas p38α phosphorylation resulted in Ras-transformation inhibition, p38γ increased Ras-dependent growth. The PTPH1/p38γ kinase/phosphatase signaling network was found to be important for oncogenic Ras activity in colon cancer cells [56,57]. Constitutive active Ras signaling appears to be frequent in cSCC [37]. 

**Figure 4:**
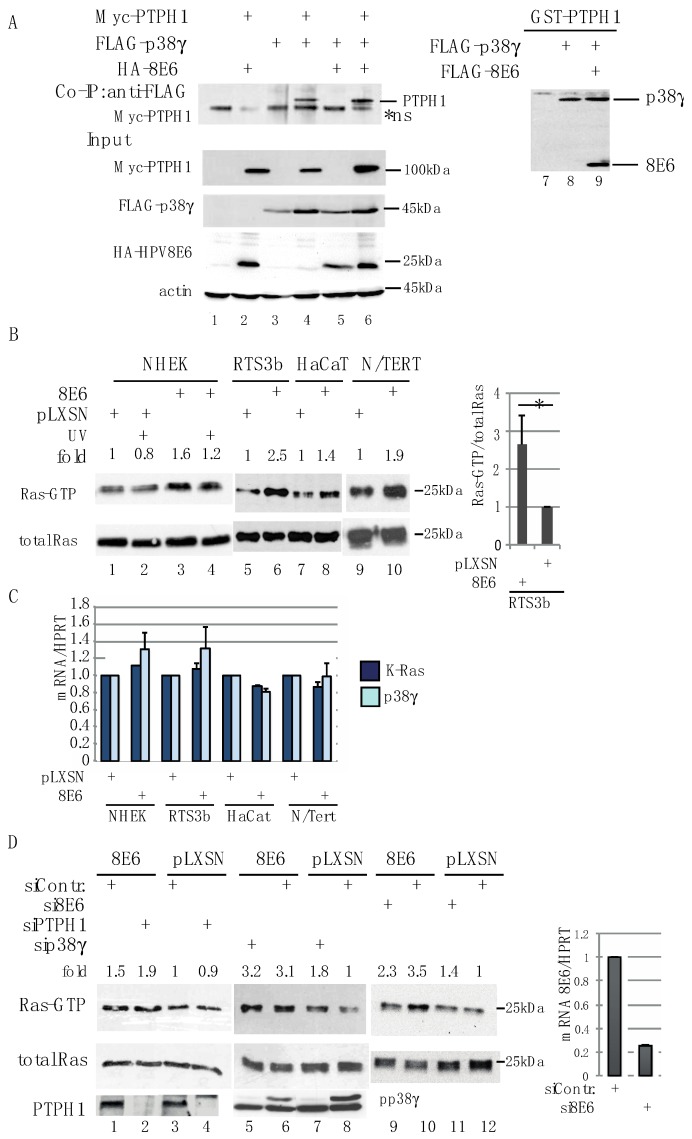
HPV8E6 expressing keratinocytes have increased level of active Ras. (**A**) Co-immunoprecipitation with extracts from C33A cells expressing recombinant FLAG-p38γ, Myc-PTPH1 and/or HA-HPV8E6 with FLAG beads, as indicated in the figure. The blots were developed with the epitope tag antibodies. In Lanes 7–9, bacterially expressed, purified GST-PTPH1 was incubated with extracts from cells expressing FLAG-tagged p38γ alone or together with FLAG-HPV8E6. The blot was developed with the FLAG antibody. (**B**) Extracts from pLXSN control or HPV8E6 positive NHEK, RTS3b, HaCaT, or N/TERT, have been incubated with GST-RBD (Ras binding domain of Raf) fusionprotein. Ras was detected in the precipitate as well as in the input by a pan Ras antibody. The graph represents the ratio of precipitated GTP-Ras versus total Ras from 4 independent experiments. The standard deviations as well as the *p*-values are indicated. (**C**) RNA was isolated from NHEK, RTS3b, HaCaT and N/TERT HPV8E6 and empty vector control cells and qRT-PCR was performed to determine the expression of K-Ras and p38γ. (**D**) RTS3b-HPV8E6 and control cells were transfected with siRNA against PTPH1 (lanes 1–4), p38γ and HPV8E6 or non-targeting control (siContr). The extracts were used in a GST-RBD pull down assay. To demonstrate the knock down of p38γ, siRNA transfected cells have been UV-irradiated 30 min prior harvesting and extracts were used in Western blot with anti-phospho MAPK antibody to detect UV-activated phopho-p38γ, which is migrating at 43kDa, while the other MAPK members migrate at 38kDa. The fold ratios between GTP-Ras and total Ras are given. RNA form RTS3b-8E6 cells transfected with si-RNA against HPV8E6 or control was used for qRT-PCR to demonstrate the efficiency of the HPV8E6 knock down.

First of all, we addressed whether HPV8E6 interferes with the complex formation between PTPH1 and p38γ. We co-expressed all three proteins with epitope tags and performed immunoprecipitations. Myc-PTPH1 co-precipitated along with FLAG-p38γ, independent whether HPV8E6 was co-transfected or not (Figure 4A), indicating that HPV8E6 does not interfere with the PDZ-mediated interaction between p38γ and PTPH1. To further confirm this, bacterially expressed and purified GST-PTPH1 protein was incubated with extracts of cells which had been transfected with expression vectors coding for FLAG-p38γ and FLAG-HPV8E6. Again, the precipitation of p38γ by GST-PTPH1 was not affected by HPV8E6 (Figure 4A). Therefore, we got no hints that HPV8E6 interferes with p38γ binding to PTPH1. 

Next, we determined the activation status of Ras in HPV8E6 expressing keratinocytes. The GST-fused minimal Ras binding domain of Raf1 (RBD) was used to precipitate active GTP-bound Ras from cell extracts [58]. The amount of GTP-bound Ras was higher in HPV8E6 expressing keratinocytes compared to the pLXSN empty vector cells. This was found in HaCaT, RTS3b, N/TERT cells as well as NHEK (Figure 4B). As already shown in Figure 1B, there was no significant difference in the mRNA expression of PTPH1 (see Figure 1B). QRT-PCR confirmed that the expression of K-Ras and p38γ did not vary between HPV8E6 expressing and empty vector HaCaT, RTS3b, N/TERT and NHEK, respectively (Figure 4C). The siRNA-mediated suppression of PTPH1 or p38γ did not affect the activation of Ras since we observed no significant reduction in the amount of GTP-Ras, neither in RTS3b-8E6 nor in control cells (Figure 4D). The elevated level of active Ras was dependent on the presence of HPV8E6, since siRNA-mediated suppression of HPV8E6 in RTS3b-8E6 cells reduced the level of GTP-Ras. The finding that the amount of active Ras was still elevated compared to pLXSN cells may rely on the fact that in spite of 80% suppression there was still considerable level of E6 mRNA present in the RTS3b-8E6 cells upon transfection of E6 specific siRNA (Supplementary Table S2). From these data we conclude that HPV8E6 increases the level of active Ras, which is independent of PTPH1.

### 3.5. PTPH1 and p38γ Are Involved in Control of the UV-Activated EGFR 

**Figure 5:**
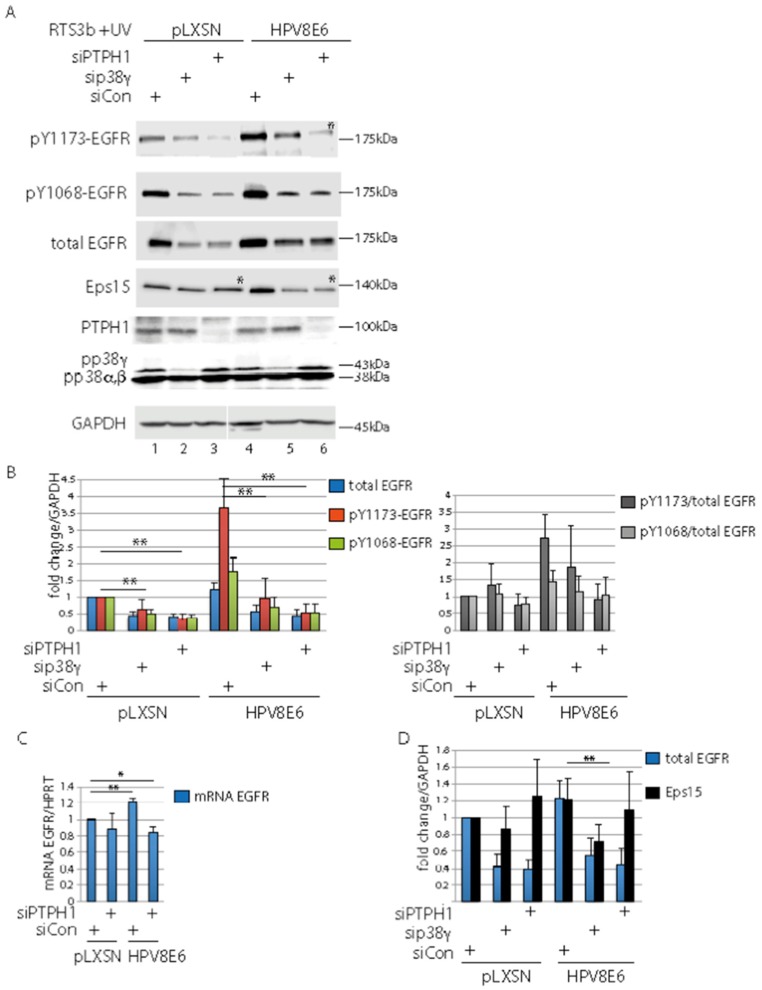
PTPH1 affects the EGFR in UV-exposed keratinocytes. (**A**) RTS3b-8E6 and control cells were transfected with siRNA against PTPH1, p38γ or control. 24 h later the cells were incubated in serum free medium for 16 h, irradiated with UV light, followed by further 30 min incubation. Total cell extracts were used in Western blot to detect the presence of the proteins as indicated in the figure. Note the higher migration of Eps15 and the pY1173-EGFR in Lanes 3 and 6, were PTPH1 was suppressed (indicated by *). (**B**,**D**). The blots were developed with the use of IRDye conjugated secondary antibodies (Li-Cor) and the signals were quantitated by the Image Studio software from Li-Cor. The signals obtained for EGFR versions and Eps15 were normalized to those for GAPDH and the fold differences were calculated. The relative amount of each factor in RTS3b-pLXSN-empty vector cells, transfected with control siRNA was set as 1. The graph shows the relative values for EGFR, pY1173-EGFR, pY1068-EGFR and the ratio pEGFR/EGFR from three and of Eps15 from six independent blots. (**C**) RT-PCR to determine the expression of the EGFR (n = 4) (** *p* < 0.01, * *p* < 0.05). The standard deviations are provided.

Previous reports demonstrated that PTPH1 dephosphorylates the EGFR at pY1173 thus controlling the stability of the EGFR [26,36,59]. Since we have shown in our previous studies that HPV8E6 modulates the RTK activity of the UV-treated EGFR we investigated whether PTPH1 is involved in this process. PTPH1 and p38γ were suppressed in RTS3b-8E6 and empty vector cells by siRNA. The cells were incubated in serum free medium overnight and were UV-irradiated 30 min prior harvesting to UV-activate the EGFR, as previously described [22]. Western blots revealed 1.8-fold higher level of pY1068-EGFR in siControl transfected RTS3b-8E6 cells compared to pLXSN-empty vector cells (*p* < 0.05), which is in line with our previous observations [22]. However, the 8E6 enhancing effect on EGFR tyrosine phosphorylation was more pronounced with regard to pY1173-EGFR, which was 3.6-fold higher in siControl E6 cells compared to empty vector cells (*p* < 0.01). The lack of p38γ and PTPH1 reduced the amount of pY1173-, pY1068- as well as the total EGFR by about 50% (Figure 5A,B). Notably, a strong reduction of the pY1173-EGFR signal occurred after the PTPH1 knock down in RTS3b-8E6 cells compared to E6 siControl cells (*p* < 00.1) (Figure 5A–C). From this, we conclude that the weaker signals detected by the antibody against pY1068- and pY1173-EGFR upon suppression of PTPH1 were not due to the loss of specific dephosphorylation but to reduced total EGFR amounts. These results imply that both, PTPH1 and p38γ, are involved in the control of the stability the EGFR. This is supported by the findings that the mRNA level of the EGFR was hardly affected by the knock down of PTPH1 (Figure 5D). Of note, upon the suppression of PTPH1 the EGFR was migrating slightly higher which is most obvious in Figure 5A, Lane 6, detecting the EGFR by the anti-pY1173 antibody with RTS3b-8E6 extracts. This is in line with the notion that PTPH1 is dephosphorylating the UV-activated EGFR, yet the phosphorylated version may be unstable in the absence of PTPH1 leading to its enhanced degradation. However, the role of the dephosphorylation event and the PTPH1 protein in the control of the UV-activated EGFR remains unclear. 

In mammalian cells, it has been shown that Eps15 is phosphorylated by the EGFR at Y849 upon EGF stimulation and that PTPH1 dephosphorylates pY849-Eps15. The tyrosine phosphorylation of Eps15 is necessary for ligand-regulated endocytosis and degradation of the EGFR [26,59,60]. Probing the blots with an antibody against Eps15 revealed slightly higher migrating bands in lanes with extracts from empty vector as well as HPV8E6 cells, when PTPH1 was suppressed (Figure 5A, Lanes 3, 5 *; Supplementary Figure S2). This is in line with the notion that the tyrosine phosphorylation of Eps15 is induced by UV light, and Eps15 is dephosphorylated by PTPH1 under these conditions. Quantifications revealed that PTPH1 is not involved in the control of the stability of Eps15, in contrast to the EGFR (Figure 5C). Merely, the knock down of p38γ in RTS3b E6 cells reduced the amount of Eps15 significantly, due to unknown reasons. Taken together, PTPH1 regulates the UV-activated EGFR in cooperation with p38γ in the keratinocyte line RTS3b. 

## 4. Discussion 

In order to ensure viral DNA replication in the differentiating epithelia, HPV induce proliferation in suprabasal differentiating keratinocytes by uncoupling cell-cycle arrest from epithelial differentiation, which is largely mediated by the HPV E6 and E7 oncogenes. They exert their biological activities by targeting cellular regulatory proteins involved in growth control [1]. Our results presented here show that HPV8E6 targets PTPH1. This interaction does not result in the inactivation or inhibition, as in the case of most HPV-targeted growth regulators, such as p53, pRb, Bak, EP300 or MAML1 [1]. By contrast, the binding of HPV8E6 to PTPH1 correlates with higher protein level, and increased PTPH1 phosphatase activity, which we have demonstrated by a phosphatase assay. 

We observed more endogenous PTPH1 in two immortalized keratinocyte lines HaCaT and RTS3b as well as in NHEK expressing HPV8E6 without detecting any difference in mRNA transcription indicating that HPV8E6 acts at a post-translational level on PTPH1. The use of skin tumors from K14-HPV8E6 or -E2 transgenic mice allowed us to confirm this observation *in vivo*. We assume that a direct interaction of HPV8E6 leads to the stabilization of PTPH1 since we regularly observed higher amounts of recombinant PTPH1 when wt HPV8E6 or point mutants were co-expressed as well as upon co-expression of HPV8E6 with fragments of PTPH1. The mechanism underlying this process yet needs to be carefully analyzed. 

In two different cell lines, RTS3b and HaCaT, and by two different approaches, the BrdU assay, which measures active DNA synthesis *in vitro*, and the proliferation assay, we demonstrate that PTPH1 supports proliferation of immortalized keratinocytes. Since DNA synthesis was reduced upon knock down of PTPH1 in empty vector as well as in HPV8E6 HaCaT cells, we conclude that the growth supporting function of PTPH1 is not due to the expression of HPV8E6 but an activity inherent to endogenous PTPH1. This is not unexpected since HPV8E6 did not modify the enzymatic activity of PTPH1, as shown in Figure 2A, but merely increased its intracellular level. A growth supporting contribution of PTPH1 was further observed by the sh-RNA-mediated suppression of PTPH1 in HPV8E6 expressing RTS3b cells. This slowed down the proliferation by about 50% in the early culture during the time from 24 h to 48 h after seeding. The fact that the sh-RNA-mediated suppression of PTPH1 retarded the proliferation of the RTS3b-pLXSN control cells in such a manner that they could not be propagated for longer period of time (data not shown) indeed hampered the analysis of empty vector cells, but is in line with the model. A functional contribution of PTPH1 in induction of cell proliferation has been previously reported in various types of cancer cells. PTPH1 was found to be over-expressed in primary human colon cancer and its depletion inhibits colon cancer growth [56]. PTPH1 was over-expressed in 49% of primary breast cancer and PTPH1 level positively correlated with metastasis [27]. Activating mutations in PTPN3/PTPH1 were shown to promote cholangiocarcinoma cell proliferation and migration and were associated with tumor recurrence in colon carcinoma patients [30]. PTPN3/PTPH1 supported tumor growth and metastasis in human glioma [29,32] and promoted resistance and stem cell like characteristics in ovarian cancers [31]. In contrast to these reports, in lung cancer cells, PTPH1 was characterized as suppressor of cell proliferation [26], pointing to a cell type or tumor specific effect of PTPH1. In correlation with these studies, data from the human protein atlas on PTPH1 expression as prognostic marker for cancer are ambiguous. In renal and endometrial cancer protein expression level of PTPH1 was favorable and in breast cancer unfavorable for patients’ outcomes (https://www.proteinatlas.org). Although PTPH1 expression had been demonstrated in SCC, a role of PTPH1 in the pathogenesis of SCC, particular as target of βHPV has to be confirmed and requires a deeper mechanistic analysis and the study of more samples to obtain significant results. 

Our data is in line with the notion that PTPH1 is among the factors that mediate the ability of HPV8E6 to stimulate proliferation and prolong the survival of keratinocytes. The growth stimulatory activities of HPV8E6 have been observed previously [20,53] and could be confirmed here (Supplementary Figure S1). The observations that HPV8E6-K64A, deficient in MAML1 binding [20] and HPV8E6Δ132-136, deficient in p300 binding [46,61], both were reported to retain their proliferating supporting activities [20,53], and both still bound to PTPH1 [23] (Figure 2B) are in line with this notion. 

Our results suggest that PTPH1 supports cell proliferation by regulating EGFR yet the underlying mechanism remains unclear. Upon the knock down of PTPH1 the level of pY1173-EGFR was reduced. This was unexpected since the lack of PTPH1 should have increased the abundance of pY1173-EGFR if PTPH1 is an EGFR specific phosphatase in our keratinocytes. Indeed, the appearance of higher migrating pY1173-EGFR related band when PTPH1 was suppressed (Figure 5, Lane 5 vs. Lane 6) is in line with a PTPH1-mediated-dephosphorylation of the UV-activated EGFR. However, the loss of PTPH1 resulted in reduced/low level of the total EGFR. Therefore, it may be conceivable that the pY1173-EGFR version is unstable in the absence of PTPH1 and will undergo enhanced degradation. Findings by Ma et al. suggested that the PTPH1-mediated dephosphorylation of the EGFR at pY1173 and not at pY1068 counteracts the proteasomal degradation of the EGFR leading to the membranous accumulation of the EGFR in breast cancer cells [36]. A role of PTPH1 independent of its phosphatase activity in the regulation of the stability of a growth factor receptor is known. PTPH1 bound the vitamin D receptor (VDR), leading to mutual stabilization of PTPH1 and VDR. In correlation, PTPH1 mediated stimulation of breast cancer cell growth was dependent on its stimulatory effect on the VDR protein [27]. The underlying mechanism of the PTPH1 and p38γ-mediated control of the UV-light activated EGFR in keratinocytes remains to be clarified. 

Our results presented here confirm our previous studies where we reported that HPV8E6 augments the level of pY1068-EGFR in response to UV-irradiation [22]. In addition, we observed here that HPV8E6 selectively enhances the EGFR autophosphorylation of Y1173 beyond that of Y1068. A dynamic of the single tyrosine phosphorylation events of the EGFR has been reported. pY1173 serves as docking site for multiprotein complexes, including Gab1 and phospholipase C gamma PLCγ which are associated with proliferation and survival, while pY1068 is a docking site for the ubiquitin ligase Cbl, initiating ubiquitination and proteasomal degradation of the EGFR (reviewed in Reference [62]). Therefore, one might speculate that by conferring selective autophosphorylation of Y1173 beyond that of Y1068 HPV8E6 might direct EGFR downstream signaling towards the proliferation and survival rather than to proteasomal degradation of the EGFR. 

Based on reports on a role of PTPH1 and p38γ in colon carcinoma cells [56,57] we analyzed the Ras protein level in E6 expressing cells. With the use of three keratinocyte lines as well as primary keratinocytes, we present for the first time that the level of active Ras is elevated in HPV8E6 expressing cells. This effect was not altered by UV-irradiation but depended on E6 expression. The observation that no significant reduction of the level of GTP-Ras was visible upon the knock down of PTPH1 or p38γ implies that activation of Ras was independent of the PTPH1-p38γ network in these keratinocytes. There are several reports pointing to enhanced Ras signaling in βHPV-induced lesions independent of oncogenic mutations. Notably, Ras mutations have been shown to be present in only a subgroup of epithelial tumors on sun-exposed skin [63]. In clinical cSCC samples obtained from melanoma patients, which were treated with the Raf-inhibitor vemurafenib, H-Ras mutations and the presence of HPV were mutually exclusive [64]. In keratinocytes derived skin tumors from untreated K14-HPV38 E6/E7-Tg mice no H-Ras and K-Ras mutations were found. Nevertheless, the MAPK-kinase pathway was activated [9]. Similar, activating mutations of K-Ras were not detected in skin tumors of mice expressing the complete genome early region of HPV8 (K14-HPV8CER) [12,65]. In these mouse studies, it had not been tested whether the expression of E6 or E7 modulates the GTP loading status of Ras. Our results demonstrating that HPV8E6 increases the level of GTP-bound Ras in keratinocytes point to an additional not yet described step by HPV8E6 to modulate keratinocyte proliferation. Data obtained with mouse models are in line with a role of Ras activation in papilloma formation. The capability of cells to respond to Ras activation differs within different epidermal compartments with the basal layer being the most sensitive [66]. Using inducible Cre mouse models, expression of oncogenic K-Ras in hair follicle and interfollicular stem cells, the cells which are assumed to be infected by HPV [13], led to papillomas whereas transduction of transient amplifying cells with active Ras cells did not [67,68]. Interestingly, it has recently been shown that in HPV8 transgenic mice papilloma formation is driven by Lrig1+ hair follicular stem cells which expand into the infundibulum and overlaying epidermis [69]. Taken these observations together, HPV8 E6-mediated activation of Ras in sensitive stem cells might contribute to the induction of proliferation. 

In summary, our results presented here suggest that HPV8E6 supports the proliferation of infected keratinocytes via targeting of PTPH1 and activating Ras. This may contribute to keep infected keratinocytes in a proliferative state, a step which is required for viral DNA amplification in the differentiating epithelium. However, this hyperproliferation provides an increased chance of acquiring additional mutations in the human epidermal stem cell compartment particularly upon UV-exposure which may then favor the progression to cSCC [36]. Our data therefore support the hypothesis that βHPV act at early stages in cSCC formation [66]. 

## Supplementary Materials

The following are available online at https://www.mdpi.com/2073-4409/8/3/244/s1, Figure S1: HPV8 sustains keratinocyte proliferation, Figure S2: PTPH1 dephosphorylates Eps15, Figure S3: HPV38E6 does not increase the level of PTPH1, Table S1: Sequences of primer used in RT-PCR, Table S2: E6 expression in keratinocytes.
