# Supplementary material for: The Protein Tyrosine Phosphatase H1 PTPH1 Supports Proliferation of Keratinocytes and is a Target of the Human Papillomavirus Type 8 E6 Oncogene
Source: Cells. 2019 Mar 14;8(3):244. doi: 10.3390/cells8030244 (PMC6468676; doi:10.3390/cells8030244)
Supplement: Supplementary file 1 [file cells-08-00244-s001.pdf]

Supplementary Tables and Figure

Gene	Fw (5´->3´)	Rev (5´->3´)	Probe
K-Ras	AAGGTGCGGGAGAGAGGCCTG	TGCCTACGCCACCAH´GCTCCA	-
HPV8E6	CCGCAACGTTTGAATTTAATG	ATTGAACGTCCTGTAGCTAAT TCA	-
HPV38E6	CAATTTCACGGCCAAAGACA	GAGGATTTTGTTTTTGCATGTTGT	-
PTPH1	TCAGCAAATGAGCGGACA	AGAACACACGCATGACCAAG	#63
MAPK12	CCTCGCCTAGCTTCTCATGT	CCTGGATGACTTCACGGACT	#9
EGFR	GCAGCATGTCAAGATCACAGA	CCAATGCCATCCACTTGATA	#3
HPRT:	CGAGCAAGACGTTCAGTCCT	TGACCTTGATTTATTTTGCATACC	#73

Supplementary Table 1: Sequences of primer used in RT-PCR. K-Ras and HPV8E6 PCR products were detected by SYBRGreen. PTPH1, MAPK12/p38γ, EGFR and HPRT amplification products were detected by the UPL probes, as indicated.

	ct-value
HaCaT-8E6	21.9
NHEK-8E6	10.5
N/TERT-8E6	14.03
RTS3b-8E6 siCon	14.13
RTS3b-8E6 siE6	15.63
RTS3b-38E6	19.99

Supplementary Table 2: E6 expression in keratinocytes RNA from the various keratinocytes expressing E6 was used for RT-PCR to determine the RNA level of E6. In each the pLXSN controls were used as well. No E6 RNA could be detected in these lines. The average ct-values from two independent experiments are given.

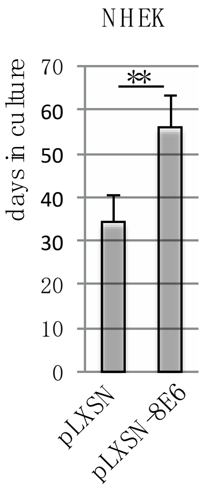

Supplementary Figure 1. HPV8E6 sustains keratinocyte proliferation. NHEK cells were transduced with recombinant pLXSN control viruses or pLXSN-8E6 (day 1) followed by selection with neomycin. The cells were cultivated and trypsinized when they reached 80% confluence. The days until they failed to reattach to the culture plate were counted. The graph represents the average number of days from 4 independent experiments. (** p< 0.05).

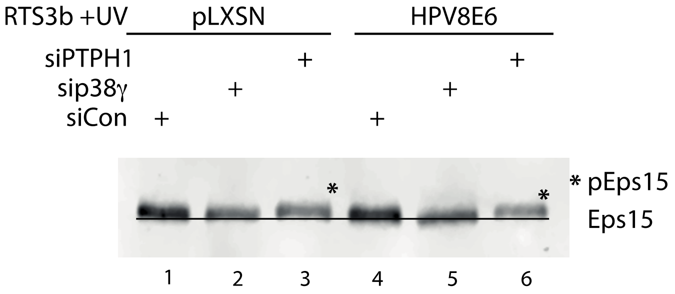

Supplementary Figure 2. PTPH1 dephosphorylates Eps15. RTS3b-8E6 and control cells were transfected with siRNA against PTPH1, p38γ or control. 24h later the cells were incubated serum free medium for 16h, irradiated with UV light, followed by further 30 min incubation. Total cell extracts were used in Western blot to detect the presence of the Eps15. Note the slightly higher migration of Eps15 in lanes 3 and 6, were PTPH1 was suppressed (indicated by*), visualized by a black line. This may represent a phosphorylated version of Eps15.

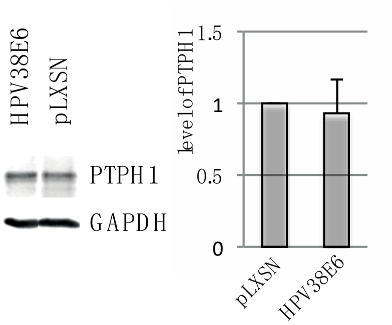

Supplementary Figure 3. HPV38E6 does not increase the level of PTPH1. RTS3b cells were transfected with pLXSN-HPV38E6 or empty vector using Fugene6 and selected by G418. Cell extracts were used for WB to detect the expression of PTPH1 and GAPDH. The graph shows the relative level of PTPH1 from 6 experiments. The standard deviation is included.
